# An alternative pathway for alphavirus entry

**DOI:** 10.1186/1743-422X-8-304

**Published:** 2011-06-15

**Authors:** Joseph P Kononchik, Raquel Hernandez, Dennis T Brown

**Affiliations:** 1 Department of Molecular and Structural Biochemistry N C State University, Raleigh, NC 27695, USA

## Abstract

The study of alphavirus entry has been complicated by an inability to clearly identify a receptor and by experiments which only tangentially and indirectly examine the process, producing results that are difficult to interpret. The mechanism of entry has been widely accepted to be by endocytosis followed by acidification of the endosome resulting in virus membrane-endosome membrane fusion. This mechanism has come under scrutiny as better purification protocols and improved methods of analysis have been brought to the study. Results have been obtained that suggest alphaviruses infect cells directly at the plasma membrane without the involvement of endocytosis, exposure to acid pH, or membrane fusion. In this review we compare the data which support the two models and make the case for an alternative pathway of entry by alphaviruses.

## Alphaviruses

Alphaviruses are transmitted to vertebrates by hematophagic insects such as mosquitoes and ticks [1]. These insects are the vectors in the enzootic cycle. Reptiles, small mammals and birds are primary reservoirs. Humans and larger mammals are largely a dead end in the virus life cycle due to the low levels of viremia produced. Symptoms of alphavirus infection vary from asymptomatic to encephalitis or arthritis [2]. Of those that cause disease, Eastern, Western and Venezuelan encephalitis viruses contribute significantly to disease in large mammals and humans[3]. Chikungunya is reemerging as a threat to humans and has been gaining ground in Africa, Asia, the Philippines and Italy with imported cases in France and in international travelers returning to the United States and South America [4-8].

Sindbis Virus (SINV), the prototype virus of genus *alphavirus *in the family *Togaviridae *is a group IV membrane containing virus with a positive sense RNA genome [9]. It is widely used in laboratory studies due to its non-pathogenic phenotype in humans and biosafety level 2 containment status. SINV can be grown to high titer in both mammalian and insect cells [10,11] and has been shown to infect diverse cell types. Certain strains of SINV, specifically the heat resistant SINV (SVHR) are ideal for Alphavirus entry studies. Purified SVHR, in contrast to other membrane containing viruses, can have a particle to plaque-forming-unit (pfu) ratio that approaches unity [11]. Knowing that every particle is infectious ensures that all observations of cell-virus interactions are of productive virus infections. These features of SINV are optimal for the study of entry of this class of membrane containing viruses including studies involving direct observation by electron microscopy. The term virus entry refers specifically to the mechanism by which the virus binds to the host cell receptor, penetrates the cell membrane barrier and releases the infectious RNA into the cell initiating the infection.

## Alphavirus Structure

**Figure 1:**
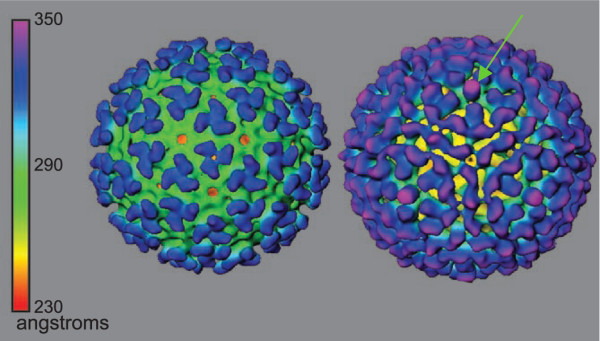
**Cryo-EM reconstruction of Sindbis Virus**. Cryo-EM reconstruction of Sindbis Virus at neutral pH (pH 7.0) and at low pH (pH 5.3) illustrating the conformational change that occurs at this threshold pH. The green arrow highlights the protrusion that appears at the 5-fold vertex at low pH. Reprinted by permission from Elsevier from: *Paredes, A. M., Ferreira, D., Horton, M., Saad, A., Tsuruta, H., Johnston, R., Klimstra, W., Ryman, K., Hernandez, R., Chiu, W., and Brown, D. T. (2004). Conformational changes in Sindbis virions resulting from exposure to low pH and interactions with cells suggest that cell penetration may occur at the cell surface in the absence of membrane fusion. Virology ****324****(2), 373-86*.

Alphaviruses are small 70 nm viruses that have 240 copies each of three structural proteins, E1, E2 and capsid (C) assembled in a 1:1:1 stoichiometry. These three proteins create two nested T = 4 icosahedral shells that sandwich a host derived lipid bilayer [12] (Figure 1). The outer protein shell is composed of E1 and E2 heterodimers that assemble into aggregates of three producing the three pronged spike which protrudes from the virus surface. The inner protein shell is made of only C, encapsulating the 49S RNA. This inner protein shell is the shape determining component of the virus [13,14]. The organization of the E1/E2 complexes on the cell membrane likely exist in a two-dimensional 6-fold symmetry sheet prior to capsid envelopment [15]. When the membrane glycoproteins begin the process of encapsulation, the nucleocapsid recruits the E1-E2 trimers into the developing outer shell by specifically binding the E2 endodomains. Through its repeated 240 interactions between a hydrophobic cleft on C and the E2 endodomain this process organizes the glycoproteins into the 6-fold and requisite 5-fold rotational arrays necessary to form a three-dimensional icosahedral structure [16]. Mutations which disrupt this process result in structurally misshaped particles [13,17,18] The resulting virus particle has 80 spikes that are primarily made of E2 (colored blue in Figure 1 pH 7.0) with a protein skirt that is primarily E1 (colored green in Figure 1 pH 7.0) which covers the incorporated membrane [19,20]. The two protein shells with their significant level of lateral and transmembrane interactions [21,22] result in a very rigid and precisely organized particle that is unlike that of other membrane containing viruses. It has been shown recently, that the particles of mammalian and insect grown SVHR are not structurally identical [23]. The cellular response to infection by insect and mammalian derived virus has also been shown to be different [24]. Particles produced from insect cells are more compact, lacking some RNA intercalation in the capsid protein shell seen in mammalian grown virus. The thickness of the membrane of virus produced from mosquito and mammalian cells does not solely account for the difference in the structure of the virus particles; however the outer protein shell seems to be extended in the mammalian grown virus suggesting that the protein organization between the two particles may be in slightly different functional conformations.

The highly organized structure of the alphaviruses with the many protein-protein interactions stabilizing the structure and the membrane bilayer occluded by the outer protein shell have important implications for the process of entry of the viral genome into host cells. Whereas many membrane containing viruses such as influenza can be described as a membranous structure with embedded proteins the alphaviruses are protein shells with an associated membrane. This protein shell must be compromised if the virus is to transfer its RNA to the cell cytoplasm. The structure of the virus protein shell may be critical for the mechanism of genome delivery to work properly. It is hypothesized that the E1 protein in the mature alphavirus exists in a metastable configuration poised to use the energy stored during virus assembly for the entry process upon encountering an appropriate trigger [25,26]. The energy stored for this conformational change is the result of folding the E1 protein though a series of disulfide bonded intermediates as the protein is compacted into the metastable structure during the assembly of the spike heterotrimer [25]. The metastable nature of the spike complex is revealed when the E1 protein is released from the mature virus using detergent because the protein reorganizes into several disulfide bridged configurations which can be separated on non-denaturing gels[25].

The current molecular model for how the virus transfers its RNA into the cell is hypothesized to be by low pH mediated membrane fusion, after endocytosis of the virus attached to its receptor. E2 contains the receptor binding sequence while E1 is known to contain the properties necessary for membrane fusion [9]. It is predicted that as the endosome is acidified, the virus membrane fuses with the endosome and releases the nucleocapsid into the cell cytoplasm. There are some reports which implicate ribosomes in the process of releasing RNA from its association with the capsid protein [27-29]. The process by which E1 mediates membrane fusion can theoretically be supported by a crystal structure of E1* (PDB code: 1I9W) which displays the position and the conformation of the putative fusion domain [30] and flexible regions within the protein. This structure was obtained by proteolytic cleavage from purified Semliki Forest Virus (SFV). A second structure was obtained by expression of the E1 ectodomain in *E. coli*. [31-33]. This crystal structure of E1 was subsequently fit into a cryoEM-reconstruction of SINV (PDB code: 1Z8Y) [34]. The crystal structure of the E1 protein has three primary domains I, II and III. Domain I is the NH proximal domain that contains the putative fusion loop. Domain II is the central domain and domain III is the most distal domain. One important caveat when interpreting structural data is that the data represents a native structure or structural intermediate of the entity in question. This condition would require that sound biochemical data confirm assumptions about the structural data without imposing other methods of confirmation to the analysis. A second condition would be that the fit of the higher resolution crystal structure not be distorted in the larger density landscape of the second much more diffuse cryoEM density. One such analysis put in question the number and location of the disulfide bonds in the native, infectious form of E1. In the crystal structure, there are a number of disulfide bonds identified; supporting the hypothesis that disulfide bonds play a role in protein assembly [35]. However some cystines identified as participating in disulfide bridges in the crystal structure were identified as free cystines using protein modification and mass spectrometry in the intact infectious virus [36]. These conflicts are not unexpected because the protein crystal structure is dependent on how the protein is purified, the crystals are grown, and the structure is refined. It is the nature of the crystal analysis process that the structure is of a protein in its lowest energy conformation. This means that the crystal structure could be any one of the various intermediate conformations assumed when E1 is extracted by detergent (described above) or expressed without its membrane spanning domain in an environment other than the endoplasmic reticulum.

A second caveat which seriously affects the quality of the data is that the entity to be studied not be distorted by the ability of proteins to be manipulated and expressed in some form in *E. coli*. There are currently crystal structures of an E1-E2 fusion protein (PDB codes 3MUU), chikungunya glycoproteins (PDB codes: 3N40;3N41;3N42;3N43;3N44;2XFB;2XFC) and their fit into the SINV and/or Semliki Forest Virus cryo-reconstruction (PDB codes: 3MUW;2XFB;2XFC). These structures were produced by expressing the ectodomains of the E1 and E2 glycoproteins connected together by a linker (58). This fusion protein was constructed due to the inability of expressing E2 in *E. coli*. While it is common practice to remove troublesome domains from proteins to allow for crystallization to occur, how is it determined when there has been too much manipulation of the sequence? These structures have the same problems as E1*. The assumption that the transmembrane domains, which have been removed in the c DNA clone, and integration into the ER membrane play no role in the correct folding and assembly of the spike structure is an unproven assumption. For virus entry of the macromolecular SINV particle, structure is critical. The overall alphavirus icosahedral structure is unique in that it is a membrane containing virus that does not have the membrane exposed. The membrane itself is not the form determining factor as with influenza, and because it is beneath the outer protein shell is not readily available to fuse with host membranes. The essential role of the membrane may be to provide the scaffold upon which the virus is assembled. To fully explore the mechanism of virus penetration and entry other methods of analysis other than interpretations of structural fit can provide the missing events required for SINV infection.

## Adsorption and receptor recognition

The process of virus attachment/absorption to the host cell is probably a multistep event. It is not a new proposal that virus infection begins with scanning of the cell surface that begins with a general "sticking" to the cell via one or more proteins (or the cell membrane itself) followed by "rolling" on the cell surface as it locates the proper receptor to initiate the penetration step of virus entry. This type of interaction has been identified recently using single particle fluorescence resonance energy transfer (FRET). SINV saturated with a membrane intercalated fluorescent self-quenching dye can be visualized when the dye is excited with a specific wavelength light [37]. Fusion of virus with the cell membrane is detected when lipid mixing (fusion) releases the proteins and dequenching of the dye occurs causing it to fluoresce. Using this technique it has been shown that SINV moves on the cell surface in a neutral pH environment. If the pH of the medium bathing the cells is lowered the virus does not fuse with the cell surface as is the case with influenza, rather the virus "freezes" in place (K. Wenniger, unpublished observation). These observations indicate that viruses probe the cell surface for the proper receptor molecule.

As arboviruses, alphaviruses infect insect and vertebrate hosts. Since alphaviruses need to infect cells which provide widely divergent biochemical and genetic environments, it is likely that they either use a ubiquitous receptor, or are able to use multiple proteins as a receptor. The receptor/s has not been identified. Many proteins and polysaccharides have been implicated as being part of the receptor complex. The list includes, heparin sulfate [38-40], the major histocompatability complex (MHC) [41,42], the major laminin receptor [43], DC sign, L sign [44], heat shock 70 protein [45], an unidentified 110 kDa nerve cell protein [46], and a 63 kDa protein in chicken cells [47]. The length of the list of possible receptors strongly suggests that there are multiple proteins that alphaviruses can use, and that the specific receptor is both cell and virus specific. If, however, there is one widely used receptor it would have to be a fundamental piece of genetic and biochemical machinery that has been conserved throughout evolution. The alphaviruses have evolved to use highly conserved proteins that span the large breath of species that serve as hosts and/or multiple proteins as a receptors.

The concept that the receptor is, at least in part, proteinaceous comes from a study which showed that protease treatment of cells prior to adsorption decreased the number of infected cells. Phospholipase and neuraminidase treatment did not have an effect on infection [48]. Using chemical cross-linking, the first candidate as a receptor was identified by Maassen and Terhorst [42] as a 90 kDa protein. Following this report there were a number of additional studies that employed various techniques to determine the receptor. One such study used soluble glycoproteins from Semliki Forest Virus (SFV) and showed that the MHC bound the glycoproteins and that detergent soluble MHC protein was able to inhibit SFV infection of HeLa cells [41]. Since then, a number of questions about the validity of this argument have arisen since cells that lack the MHC complex are not resistant to SFV infection [49]. Additionally, mosquito cells, which fundamentally lack a human immune system and thus do not express MHC, are also readily infected by alphaviruses.

The use of anti-idiotypic antibodies as receptor locators has been used as well to determine the receptor for alphaviruses. This approach is responsible for the discovery of the 63 kDa chicken protein [47] and eventually led to the implication of the high-affinity laminin receptor [43]. While this is considered a major receptor for alphaviruses, reexamination of the original experiment that identified the 63 kDa chicken protein revealed that this protein was not the chicken laminin receptor [47]. Additionally antibody against the chicken laminin receptor did not inhibit infection significantly (< 10%). This suggests that although the laminin receptor is conserved across many species, it is not the only virus receptor. It is entirely likely that there are multi-protein complexes that are not required for, but enhance infection. Other investigations of the laminin receptor used anti-idiotype antibodies to examine the laminin receptor as a possible virus receptor in mosquito cells. In these studies a 32 kDa protein was discovered in mosquito cells to which Venezuelan Equine Encephalitis Virus (VEE) bound as did laminin and SINV [9]. Antibodies were also used to investigate virus binding with a strain of SINV which was selected to be a rapid penetrating virus [50]. Upon binding to the cell at neutral pH assumingly to its receptor, SFV was shown to go through conformational changes as new antibody epitopes were exposed on the surface of the virus [51]. This is presumably related to the conformational change that is seen in reconstructed virus particles which were exposed to low pH [12].

## A measure of successful virus entry

There are numerous techniques that can be used to examine the interactions between the virus and the cell. These techniques can generally fall into two categories: direct and indirect observation. Direct observation uses familiar techniques such as thin-section microscopy, cryo-electron microscopy, tomography, and other less familiar techniques like freeze-fracture immunolabeling to examine virus-cell interactions. When properly prepared and processed, the interactions between the virus and cell seen in the microscope are the actual interactions that occurred at the time of fixation. By examining different times during the infection process, the possible mechanism and pathway of virus entry can be elucidated. Unfortunately, thin-sections and other direct observation techniques cannot distinguish between a successful infection process, and that of an unproductive interaction. This makes microscopy very subjective and is the primary problem when observing virus-cell interactions, as even with a relatively low particle/pfu ratio of 1:10, most observations are of virus-cell interactions that do not lead to a successful infection. For SINV, the SVHR strain can be purified to a particle/pfu ratio of 1:1, (28) and this virus was used in EM studies of virus infection that led to the direct cell penetration hypothesis of virus entry [12]. Many of the previous observations that have been made using EM are of viruses preparation made with strains or samples with high or undetermined particle/pfu ratio and therefore are difficult to interpret.

When direct observation is not possible, or cannot answer the question being asked, techniques that take advantage of indirect or secondary reporters have been used. Some indirect reporters that have been used as a measure of successful virus entry include virus RNA production [52], virus protein synthesis [53] and virus production [54]. While these tools are useful and have shed light on many virus-cell interactions, they are at a disadvantage insomuch that they cannot determine if a virus has not entered the cell as the events assayed are not entry. Many of these biochemical reporters are significantly downstream of the initial events of virus adsorption and entry. As a result, each step between entry and the reporter has the potential to be inhibited giving a false negative. To give a time-scale to RNA translation and protein synthesis, super infection inhibition is detectable 15 minutes post infection [55,56]. This implies that entry is a very fast process, and that the genome is quickly unpacked and rapidly available for processing. The speed of infection, the problems of indirect reporters, and the limits of direct observation need to be carefully considered when building an assay to measure successful virus entry.

## Alphavirus entry

Once the virus has bound to its receptor, the virus outer shell must compromise the plasma membrane barrier. The classical mechanism of membrane containing viruses to breach this barrier is by triggering fusion of the virus membrane with the host cell membrane, releasing its contents into the cytoplasm [57]. This is accomplished by a conformational change that can occur by receptor recognition as with HIV [58,59], or by environmental changes such as pH or artificially with heat or reducing agents as seen with SINV [60,61]. The conformational change of SINV was visualized using cyro-electron microscopy reconstruction of particles exposed to low pH [12]. The change resulted in a protruding spike from the 5-fold axis of the virus, compared to the lack of this density in the untreated particles (Figure 1, green arrow). The location of the spike suggests that it is both 5-fold in symmetry, and that it is likely composed of E1, the glycoprotein that expresses the putative fusion loop. Interpretation of these data in the context of the prevailing infection by fusion model would predict that the virus and cell membranes fuse and the nucleocapsid is delivered to the cell cytoplasm. However, evident from these reconstructions is that no lipid is exposed at high pH, or upon return of the particles to low pH. This observation forms the basis of the following hypothesis which suggests that there is no *a priori *reason to conclude that because a virus has a membrane, penetration must be by fusion. Discussed here are two suggested modes of entry for the alphavirus and the supporting data.

### Viral Endocytosis followed by membrane fusion

The most commonly accepted mechanism of entry for alphaviruses is via endocytosis and the subsequent acidification of the vesicle leading to membrane fusion. This is similar to the mechanism accepted for influenza. The studies in support of this mechanism are extensive, and rely on the observations that alphaviruses have membrane fusion capabilities, and that inhibition of the acidification of endosomes inhibits the entry of the virus. The original fusion assays were done with cultured cells, and it was shown that treating cells with adsorbed virus to a brief low pH environment, resulted in cell-cell fusion (fusion from without) [62]. This suggested the acid environment of an endosome could provide the requirements for fusion and penetration. However in these studies the cells were always returned to neutral pH before fusion was seen and it was subsequently shown that this return to neutral pH was required to induce fusion of the virus and cell membranes[63]. The pH of the endosome does not return to neutrality. The analysis of fusion was then moved from a live cell culture to an in vitro assay including liposomes and other artificial membranes [64]. In these assays, the fusion process did not require a return to neutral pH, but required a significant amount of cholesterol in the target membrane [65]. This cholesterol requirement was supported by a report that cells grown in serum treated with Cab-O-Sil, a silicate that removes cholesterol from the serum could not be infected as measured by immunofluorescent antibody labeling of newly folded virus protein [66]. Here a third caveat must be add must be made, that is how far afield from the native system can the experimental design be taken before the results are valid biochemically but cannot reproduce the biological environment? This should especially be considered in the context of a biological macromolecule expressing multiple required functions. There are two specific concerns regarding the cholesterol requirement for infection that have been neglected. First, the liposome is protein free and therefore receptor free and second the amount of cholesterol required to induce fusion of SINV to liposomes is high, up to 50 mol%. However, the requirement for such a high concentration of cholesterol is in direct conflict with the fact that alphaviruses infect and assemble in mosquitoes, which are cholesterol auxotrophs [67,68]. Second, Cab-O-Sil, while removing cholesterol also removes a significant number of other lipids, which may be required for proper function of the cells and virus stability [68]. In fact, growing the same cell type used in the Cab-O-Sil experiment in serum-free media resulted in no significant change in their sensitivity to infection [68]. Together, these data imply that a large amount of cholesterol is not required for alphavirus infection but is required for fusion with liposomes. Thus, if cholesterol is not required for infection and fusion of living cells does not occur at acid pH the liposome model for studying virus penetration may not be valid.

Treatment of cells with chemicals that inhibit the acidification of endosomes has been used to assay the role of acid environment in the infection of mammalian cells by alphaviruses [69,70]. The assays for successful penetration were virus RNA or protein production or the production of progeny virions. It was concluded from these studies that the mechanism of entry was via endocytosis and endosome acidification. Drug studies suffer from the fact that no drug is specific for a single target and secondary and tertiary effects can affect the observation made. Because of this inherent problem it is critical that all possible controls be done and that the results are interpreted with caution. Drugs such as Bafilomycin A1, chloroquine, monensin and ammonium chloride (NH4Cl) were used to inhibit endosome acidification [69-72]. The initial intent of these experiments was to demonstrate what the effect of the lack of acidification using these chemicals would have on the infection process. It was found that virus, virus RNA, or virus protein were not produced when these agents were present during the period of virus entry and this was interpreted to indicate that lack of acidification inhibited the release of the nucleocapsid from the endosome into the cell cytoplasm. These early experiments led to the current model of alphavirus infection by low pH mediated membrane fusion. However upon more rigorous analysis it was discovered that these drugs have various inhibitory effects on virus production not directly related to virus penetration. Chloroquine was shown to reduce the amount of virus produced in mammalian cells, however in insect cells the virus titer was enhanced [73,74]. Chloroquine did increase the pH of mosquito cell endosomes as the cells were protected from diphtheria toxin which is known to require an acid environment to enter cells. Also in mosquito cells ammonium chloride was shown to inhibit steps in the non-structural protein processing after initial translation of the incoming RNA; many steps from the initial virus entry which had to have occurred [74]. While chemical inhibitors have been invaluable in the study of cellular metabolic pathways, it is imperative that the design of the experiment incorporate appropriate controls for secondary effects.

Using a SINV construct that contained a GFP reporter, a thorough study was done to assess the effect of Bafilomycin A1 (BafA1) on the entry of alphaviruses into mammalian and mosquito cells [75]. The results of this study showed that BafA1 did not prevent virus entry into mammalian or mosquito cells or inhibit RNA synthesis. It was also shown that BafA1treatment produced the same results when cells were transfected with virus RNA a process that bypassed the entry events. Additionally, the effect of BafA1 in mammalian cells was reversible and the authors concluded that BafA1 was inhibiting the proper folding of the newly synthesized proteins and that the functioning V-type ATPase was required for proper protein assembly. When examining the effect of BafA1 on mosquito cells, no observable inhibition of virus entry was observed, even at high concentrations of BAfA1. It has been demonstrated that the insect V-ATPase is sensitive to BAFA1 at nanomolar concentrations [76]. All of the data supporting entry at the plasma membrane correlate well with a recent publication showing SINV replication complexes at the plasma membrane [77] as it would be easiest to explain the presence of the replication complexes here if the mechanism of entry were at the plasma membrane.

### Entry in the absence of membrane fusion

**Figure 2:**
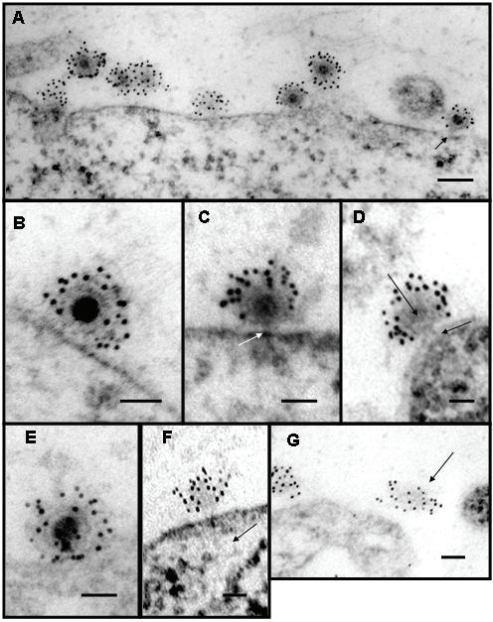
**Electron micrographs of thin sections of Sindbis virus-cell complexes at pH 7.2**. (A) Low magnification showing ''full'' and ''empty'' particles and a particle attached by a pore to the cell surface (arrow). (B) A virion attached to the cell surface before pore formation. (C) A virion with an electron dense core attached to the cell surface by a pore structure (arrow). (D) The pore at the vertex (V) of the protein shell penetrates the cell membrane (arrow). The virion has reduced electron density in the core region. (E) Reorganization of virus RNA into the developing pore. (F) An empty particle with a possible RNA molecule entering the cell (arrow). (B) An empty virion that has lost structure. Magnification scale bar (A) = 1000 A °, all others = 500 A °. Reprinted by permission from Elsevier from: *Paredes, A. M., Ferreira, D., Horton, M., Saad, A., Tsuruta, H., Johnston, R., Klimstra, W., Ryman, K., Hernandez, R., Chiu, W., and Brown, D. T. (2004). Conformational changes in Sindbis virions resulting from exposure to low pH and interactions with cells suggest that cell penetration may occur at the cell surface in the absence of membrane fusion. Virology ****324****(2), 373-86*.

An alternative mechanism of entry that is gaining supporting evidence is virus entry at the plasma membrane in the absence of endocytosis, exposure to low pH and membrane fusion. This mode of entry is proposed to employ a pore complex made from virus and host proteins that connects the interior of the virus to the host cell cytoplasm via a protein channel. Supporting this mechanism of infection is that infection by alphaviruses has been shown to be a leaky process allowing passage of ions and small molecules across the compromised plasma membrane [78-81]. The infected cell membrane becomes leaky early in the infection prior to protein expression and also late during infection [82,83]. Membrane fusion by contrast is a non-leaky process [84]. Were the viruses merely to bind to its receptor and then be taken into the cell, as is the case in the low pH -endocytosis model there would be no loss of continuity in the plasma membrane. It was shown that infection with alphavirus created pores in the plasma membrane that could be blocked with rare earth ions which allowed investigators to assign a size to the pores [81]. It has also been shown that the pores created in the infection process are large enough to allow the passage of the toxin alpha-sarcin into the cell (42). The 17 kDa, 150 amino acid protein was shown to co-enter during infection. Interestingly, it has been shown that the 6 k protein which links PE2 to E1 in the structural polyprotein can also produce pores in the plasma membrane late in infection [85] In 2004, Paredes *et al. *were able to show full and empty particles (Figure 2) on the surface of mammalian cells using thin-sections of BHK cells infected with SVHR which had a particle/pfu ratio of ≈1. Empty particles were identified by using anti-SINV antibodies with secondary antibodies conjugated to gold beads, as they would have been easily overlooked otherwise (Figures 2A, D, F). From the thin-sections, multiple particles with varying electron dense cores were shown and suggested that the virus was losing its RNA to the cell cytoplasm through a proteinaceous connection between the virus and the cell. The virus subsequently appeared to lose structural stability and was seen released from the cell leaving the proteinaceous pore behind. (Figure 2B, E, F, G). The process of cell penetration was found to be extremely fast requiring a complex process of attaching virus at 4° 0 and then adding warm media that contained fixative to capture the image. It was found that even at low temperature 4-5% of the attached particles had lost their electron dense RNA core during the binding and fixation period. Cells treated with warm fixative had 25-26% of particles which had lost their RNA. Alphavirus entry in the absence of endocytosis was also shown by infecting cells at low temperature a condition under which membranes cannot fuse and endocytosis is inhibited [86]. These experiments demonstrated that entry could occur when fusion was not possible showing the two processes to be unrelated. These data support the mechanism proposed in Paredes *et al. *which show physical evidence that alphaviruses use a proteinaceous pore to infect cells.

Additional evidence supporting alphavirus entry via a pore at the plasma membrane was obtained using freeze-fracture (Kononchik, Vancini and Brown, Virology, in press). In these experiments, high MOI of SVHR was adsorbed onto mosquito cells for half an hour after which the virus was cross-linked to cell surface proteins with gluteraldehyde. These samples were then processed via a freeze-fracture method to examine the plasma membrane and its components. The technique was a modification of the FRIL (freeze-replicate-immunolabel) technique developed by Fujimoto [87,88]. In brief, the cross-linked samples were flash frozen in liquid ethane, fractured at low temperature and pressure. The fracture plane that goes through the sample follows the path of least resistance, which is the middle of lipid bilayers making two replicas: the inner leaflet, or P-face, and the outer leaflet, the E-face. As a result, when the fracture hits a cell, it tends to follow the plasma membrane. This makes it ideal for examining proteins and interactions that occur on the cell surface. In this experiment, the replicas had cross-linked virus proteins attached to the outside of the E-face. Using immunolabeling, these proteins were labeled, and the organization of the proteins was examined by electron microscopy. The data collected showed labeled virus particles adsorbed to the cells' surface. These particles were observed as discrete rings of gold bead tags surrounding a structure that traversed the membrane bilayer. We have suggested that this transmembrane structure comprises part of the pore complex.

## Summary

Direct observation of alphavirus-cell interaction by electron microscopy and through the use of chemical inhibitors and interaction with artificial membranes has created seemingly conflicting data. In many direct observations, virus can be seen in endosomes inside the cell [89]. These data suggested that the virus particles enter through an endocytic pathway. Coupled with the conformational changes that occur upon exposure to low pH and the fusion capabilities shown by SINV, the suggested route of entry is similar to that of influenza. However, other observations including thin-sections and freeze-fracture have shown that SINV can infect without endocytosis and acidification. These studies also found that as the particle to PFU ratio of the virus employed improved to unity no virus was seen in endosomes and empty particles were seen on the cell surface. These data suggest that the mechanism for virus entry is at the cell surface and involves direct release of the virus genome into the cytoplasm by a pore-like structure.

The use of chemical inhibitors of endosome formation and acidification has relied on reporter events which occur many steps away from the process of entry. Many of the agents have secondary effect on events that occur after entry which have led to erroneous conclusions.. Likewise studies of virus interaction with artificial membranes have produced a set of requirements for the fusion event that are not related to those occurring with living cells. It appears that, although alphaviruses possess the ability to fuse membranes this is is an event that occurs under special laboratory conditions and may be unrelated to the true entry process.

Arthropod borne viruses are significant sources of disease in man and domestic animals and some are potential agents of bioterrorism. Strategies to control infections by these agents include the development of compounds which will block critical steps in the entry pathway. An accurate image of this pathway and the identification of the cell receptor participating in the entry process is critical for the development of agents which can block infection.

## Competing interests

The authors declare that they have no competing interests.

## Authors' contributions

JK, RH and DB contributed to and edited the manuscript. All authors read and approved the final manuscript.
